# Remarkably High Effective Mobility of 301 cm^2^/V·s in 3 nm Ultra-Thin-Body SnO_2_ Transistor by UV Annealing

**DOI:** 10.3390/nano16020133

**Published:** 2026-01-19

**Authors:** An-Chieh Shih, Yi-Hao Zhan, Albert Chin

**Affiliations:** Department of Electronics Engineering, National Yang Ming Chiao Tung University, Hsinchu 300, Taiwan

**Keywords:** effective mobility, transistor, SnO_2_, UV annealing, ultra-thin body

## Abstract

At an ultra-thin 3 nm SnO_2_ channel thickness, a record-high effective mobility (µ_eff_) of 301 cm^2^/V·s, field-effect mobility (µ_FE_) of 304 cm^2^/V·s, and a sharp subthreshold swing (*SS*) of 201 mV/decade are achieved at a high carrier density (N_e_) of 5 × 10^12^ cm^−2^. These excellent transport properties are attributed to ultraviolet (UV) light annealing. The resulting µ_eff_ is significantly higher than that of Molybdenum Disulfide (MoS_2_) and Tungsten Diselenide (WSe_2_), and is more than twice that of single-crystalline Si channel transistors at the same quasi-two-dimensional (2D) thickness of 3 nm (equivalent to five monolayers of MoS_2_). UV annealing not only enhances µ_eff_ and µ_FE_ but also sharpens the *SS*, which is crucial for low-power operation. This improved *SS* is attributed to reduced scattering from charged interface traps, as supported by µ_eff_-N_e_ analysis, thereby increasing the transistor’s mobility. The realization of such high-mobility devices at a quasi-2D thickness of only 3 nm is of particular importance for the further downscaling of ultra-thin-body transistors for high-speed computing and monolithic three-dimensional (M3D) integration. Furthermore, the wide bandgap of SnO_2_ (3.7 eV) enables operation at relatively high voltages, paving the way for pioneering ternary logic applications.

## 1. Introduction

Over the past decades, tremendous efforts have been devoted to sustaining Moore’s law. To lower the DC leakage current from the gate electrode, high-κ/metal-gate technology has been implemented to replace the ultra-thin SiO_2_ gate dielectric. To improve the short-channel effect and source–drain leakage current, the ultra-thin-body Fin Field-Effect Transistor (FinFET) has been used at the 22 to 3 nm technology nodes. However, as FinFET devices continue to scale into the sub-3 nm regime with thinner fin widths, fundamental challenges arise. Issues such as surface scattering, threshold voltage variation, quantum confinement, and degraded mobility have become increasingly significant [1,2,3,4,5]. To address these concerns and enable further downscaling to the 2 nm node and beyond, ultra-thin-body (UTB) transistors have emerged as highly promising candidates for monolithic 3D (M3D) integration. This is because their superior gate electrostatics can effectively suppress short-channel effects, and, when combined with M3D architecture, they enable a higher transistor density and drive current than that of FinFETs through channel stacking.

Nevertheless, as scaling approaches the deep sub-1 nm technology node, the Si channel struggles to maintain sufficient performance. At this stage, two-dimensional (2D) semiconductors emerge as promising candidates [6,7,8,9,10,11,12,13,14]. Several 2D materials, such as Molybdenum Disulfide (MoS_2_) and Tungsten Diselenide (WSe_2_), feature atomically thin channels where carriers are confined within a few atomic layers, leading to excellent gate control and an extremely low off-state leakage (I_OFF_). The absence of dangling bonds at the surface enables superior electronic properties even at monolayer thickness. In addition, their tunable band structures and appropriate bandgaps are advantageous for suppressing leakage power [15,16,17]. However, 2D materials also face severe challenges: the coulomb impurities (CIs) from the gate dielectric dominate the carrier scattering at room temperature, leading to a severe degradation of mobility [18]. Experimentally measured mobilities remain far below predictions [1,19,20,21]. Furthermore, the synthesis of 2D materials is difficult—typically requiring high growth temperatures (>600 °C) on non-Si substrates such as copper or sapphire—followed by transfer processes that are prone to defect generation, significantly lowering the mobility [22,23,24,25,26,27,28,29]. Achieving the large-scale, defect-free, and uniform growth of 2D materials on 12-inch wafers remains a major bottleneck.

On the other hand, high-mobility oxide semiconductors offer distinct advantages, including low-temperature processing, low cost, and large-area manufacturability, along with their inherently wide bandgaps, making them well suited for low-power operation [30]. Among them, amorphous oxide semiconductors such as InGaZnO have already been successfully adopted in commercial displays, benefiting from their high mobility, high uniformity, transparency, and compatibility with cost-effective manufacturing [31,32,33,34,35,36,37]. Compared with 2D semiconductors like MoS_2_ or WS_2_, oxide semiconductors offer significant advantages in terms of large-area uniformity, low-cost processing, and direct integration with Si wafers [33,38,39]. Despite these merits, when the channel thickness is scaled down to the sub-5 nm regime, oxide Field-Effect Transistors (FETs) also suffer from interface trap density (D_it_) and enhanced surface scattering, which deteriorate the device mobility [40,41].

To overcome such limitations, tremendous efforts have been devoted to enhancing the transport properties of oxide semiconductors by material engineering and structural design. Strategies such as alloy composition optimization [35,42], heterostructure engineering [43,44], and defect passivation [45,46] have been explored to improve device performance, yet the realization of ultra-thin oxide channels with both high mobility and manufacturability remains unclear. Previously, advanced oxide FETs achieved a mobility comparable to single-crystal Si nFETs at equivalent body thicknesses of 5 and 7 nm [47,48]. In this work, we further demonstrate 3 nm thick UTB SnO_2_ n-type FETs (nFETs) exhibiting record-breaking performance, with effective mobility (µ_eff_) and field-effect mobility (µ_FE_) reaching 301 and 304 cm^2^/V·s, respectively, at a carrier density (N_e_) of 5 × 10^12^ cm^−2^. The large N_e_ of 5 × 10^12^ cm^−2^ is the standard value for µ_eff_ comparison, which is required to deliver a high output current to drive the circuit at a high speed. The high µ_eff_ and µ_FE_ are further supported by the sharp subthreshold swing (*SS*) of 201 mV/decade for low gate voltage (*V_gs_*) and low power operation. At the same 3 nm thickness, the µ_eff_ value of our devices outperforms those of both conventional 2D semiconductors [1,49,50,51] and other oxide semiconductors [32,43,47,52,53]. The ultra-thin 3 nm SnO_2_ equals a five-monolayer thickness of MoS_2_, with a monolayer thickness of 0.65 nm [48,54]. Remarkably, the µ_eff_ is more than 2 times higher than that of single-crystal Si nFETs at the same body thickness of 3 nm [1,55], which allows us to further downscale the high-performance UTB FET to sub-1 nm technology nodes. The comparison of µ_eff_ is shown in Table 1. Furthermore, our SnO_2_ UTB devices can be fabricated on 12-inch wafers using a simple sputtering process, enabling low-cost and large-scale production [56,57].

In addition, SnO_2_ possesses a significantly higher phonon energy for higher temperature operation [58], much better than that of Si nFETs. Such higher temperature operation is unavoidable in AI chips, where typical power consumption can reach up to 1000 W. Moreover, because its wide bandgap (3.7 eV) is much larger than that of Si (1.1 eV), the device can also sustain high-voltage operation [58,59], thereby making SnO_2_ FETs particularly promising for new leading-edge ternary logic. These combined factors render SnO_2_ more advantageous than other oxide semiconductors and even Si transistors. Since the transistor’s effective mobility µ_eff_ is proportional to the slope of drain-current versus drain-voltage (*I_ds_*-*V_ds_*) curves in the linear region, the higher µ_eff_ results in the lower *V_ds_* and lower dynamic operation power consumption of *C_gs_V_ds_*^2^*f*/2 that successively lowers the critical chip temperature in a high-density chip. Here, *C_gs_* is the gate capacitance in an FET and *f* is the operation frequency. Minimizing power consumption is more crucial than ever, where power/energy consumption has become a critical issue for the rapid growth of AI accelerators and high-performance computing. With these advantages, SnO_2_ UTB transistors are highly promising for low-power applications and monolithic three-dimensional (M3D) integration [60], offering a practical pathway to extend CMOS scaling beyond the 1 nm node.

**Table 1:** Performance comparison of different material FETs at ultra-thin thickness.

Reference	Channel Material	N_e_ (cm^−2^)	µ_eff_ (cm^2^/V·s)
	SnO_2_ (this work)	5 × 10^12^	301 @ 3 nm
[50]	MoS_2_	-	70 @ five layers
[49]	MoS_2_	5 × 10^12^	130 @ 2 nm
[61]	WSe_2_	2.5 × 10^12^	110 @ three layers
[43]	ZnO	-	100 @ 13 nm
[62]	IGZO	-	19 @ 3 nm
[55]	Si	5 × 10^12^	70 @ 2 nm

## 2. Materials and Methods

**Figure 1:**
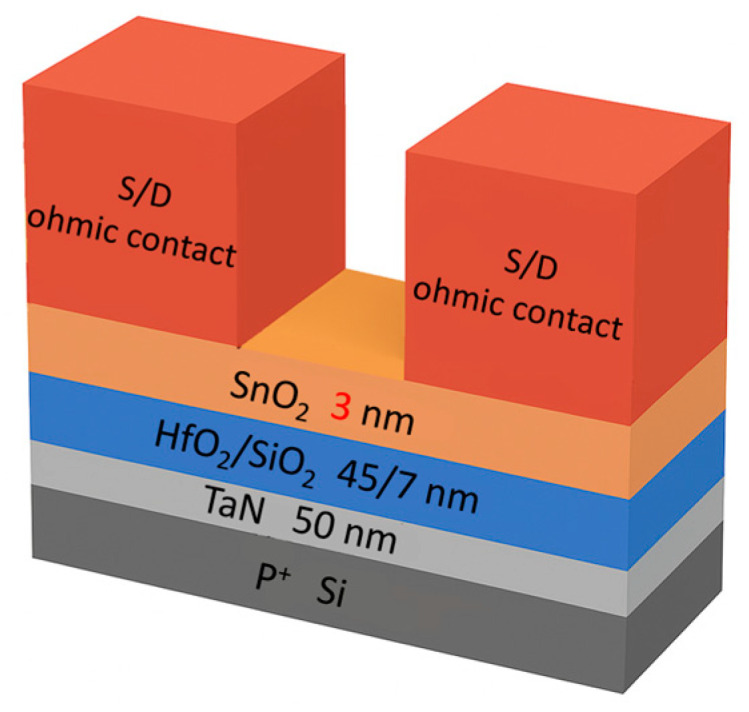
Schematic diagram of bottom-gate SnO_2_ nFET.

In this study, a standard 4-inch p^+^ Si substrate was used. A 50 nm TaN layer was first deposited by reactive sputtering to serve as the bottom electrode, with Ar and N_2_ flow rates of 100 and 10 sccm, respectively, under a DC power of 800 W. Subsequently, a HfO_2_/SiO_2_ bilayer (45/7 nm) was deposited through electron-beam evaporation as the gate dielectric stack. Post-deposition furnace annealing was performed at 425 °C for 30 min in N_2_ ambient to lower dielectric defect densities. After annealing, a 3 nm SnO_2_ channel layer was deposited through reactive sputtering using a Sn target at 25 W, with Ar and O_2_ flow rates of 24 and 25 sccm, respectively, at a chamber pressure of 7.6 × 10^−3^ Torr. The SnO_2_ was subsequently annealed in an O_2_ ambient at 425 °C for 30 min to improve its quality. The source/drain (S/D) electrodes were then fabricated by electron-beam evaporation, followed by UV annealing at 254 nm (79 mW/cm^2^) and 185 nm (11 mW/cm^2^) for 15 min. The resulting devices exhibited a channel length of 50 μm and a width of 500 μm. The materials used in the experiment were sourced from Guv Team International Co., Ltd., Taipei, Taiwan. A similar oxide ZnO FET at a 3 µm gate length showed good performance [63]. The UTB can effectively suppress the channel effect that is crucial for FinFET [64] and nanosheet FET. The *I*–*V* characteristics were measured using an Agilent HP 4155B semiconductor parameter analyzer, and *C*–*V* characteristics were obtained using an Agilent E4980A precision LCR meter (Agilent, Santa Clara, CA, USA). The gate dielectric was deposited using an E-Gun evaporator (EBX-10C, ULVAC, Tokyo, Japan) and the channels were formed through sputtering system (British Microvac 450CB, Birmingham, Ion Tech. Ltd., London, UK). Figure 1 shows the schematic diagram of the fabricated SnO_2_ FETs.

## 3. Results

**Figure 2:**
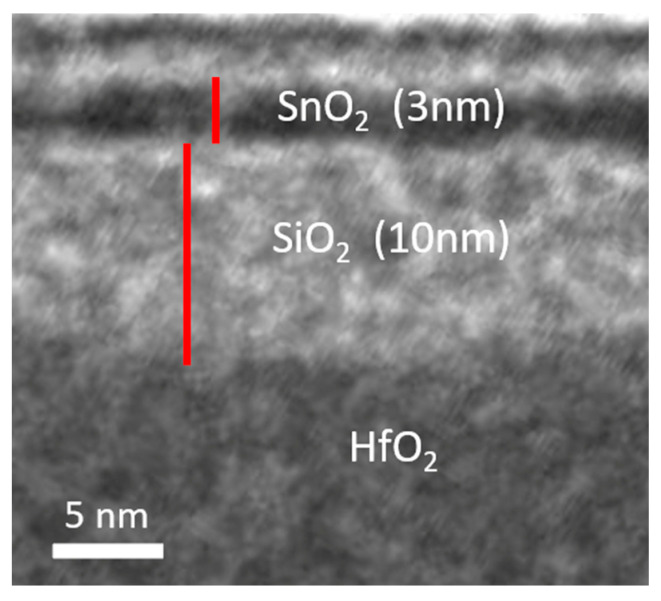
Cross-sectional TEM image of the 3 nm SnO_2_ nFET structure; the red line indicates the scale bar for 3 nm and 10 nm.

**Figure 3:**
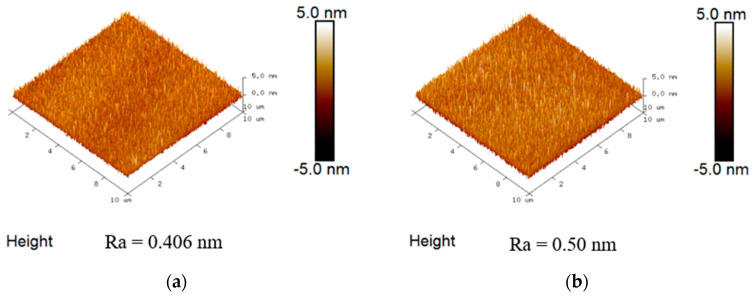
AFM images of (**a**) HfO_2_/SiO_2_ surface and (**b**) HfO_2_/SiO_2_/SnO_2_ surface.

Figure 2 presents the cross-sectional transmission electron microscopy (TEM) (JEM-2010F, JEOL, Tokyo, Japan) image of the fabricated device. The TEM clearly reveals the HfO_2_/SiO_2_/SnO_2_ stack, where the 3 nm SnO_2_ channel is comparable to 5 atomic layers of 2D MoS_2_. To mitigate scattering effects in the channel, which strongly influence µ_eff_, µ_FE_, and *SS*, a 10 nm SiO_2_ interlayer was inserted between the high-κ HfO_2_ and SnO_2_ [65,66,67]. Furthermore, subsequent UV annealing effectively reduced the interface trap density. Figure 3 shows the surface roughness data measured through Atomic Force Microscopy (AFM) (D3100, Bruker, Billerica, MA, USA) without and with the SnO_2_ channel. The smooth surface roughness is part of the reason for achieving high µ_FE_ and µ_eff_ data. Consequently, the µ_FE_, µ_eff_, and *SS* are significantly improved, thus further enhancing the overall device performance.

**Figure 4:**
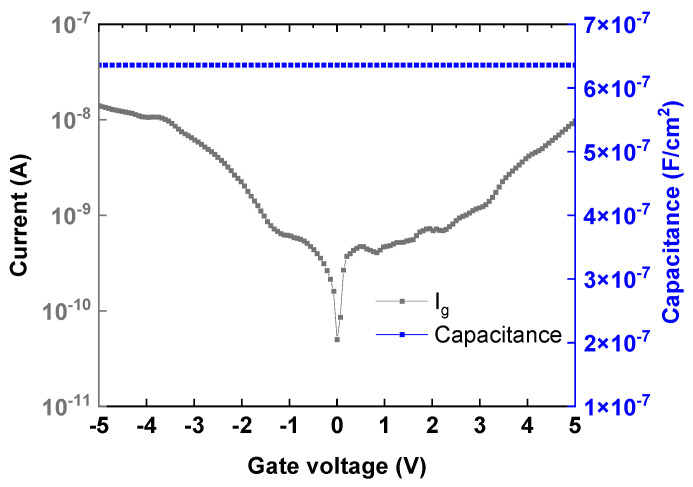
*C*–*V* and *I*–*V* of MIM capacitor.

Figure 4 shows the *I*–*V* and *C*–*V* characteristics of the gate metal/insulator/metal (MIM) capacitor with a gate dielectric stack of 7 nm-SiO_2_/45 nm-HfO_2_. A high capacitance density of 6.36 × 10^−7^ F/cm^2^ was measured at 1 kHz, and the leakage current remained below 1 × 10^−8^ A/cm^2^ at ±3 V, which is atttibuted to the thick high-κ gate dielectric.

**Figure 5:**
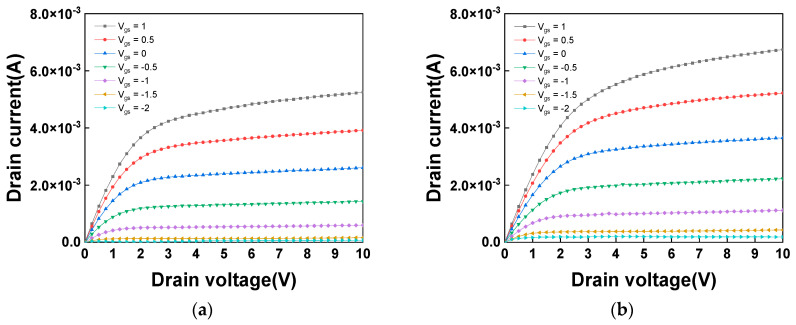
*I_ds_*-*V_ds_* characteristic for 3 nm SnO_2_ nFETs (**a**) before and (**b**) after UV annealing.

Figure 5a,b present the *I_ds_*-*V_ds_* characteristics for 3 nm thick SnO_2_ nFETs with and without UV annealing. Both devices exhibit clear pinch-off and saturation behaviors. A comparison reveals that, after UV annealing, the slope in the linear region becomes sharper and the I_D_ is higher at the same gate voltage (*V_gs_*). This improvement is attributed to the reduction in trap density by UV annealing, which in turn enhances the µ_eff_ [68]. Furthermore, the capability to operate at a high drain voltage *V_ds_* of 10 V is particularly advantageous for ternary logic, a promising architecture for future high-performance ICs. This high voltage tolerance, extending up to 20 V [58], is attributed to the material’s wide bandgap of 3.7 eV (1.1 eV for Si). Such a wide operating window is crucial for reliably defining distinct logic states at 3.3, 1.65, and 0 V. The ternary logic has unique merits of better mathematical efficiency, much decreased interconnect complexity, smaller chip size, and lower operation power consumption, all of which are urgently needed for AI chips.

**Figure 6:**
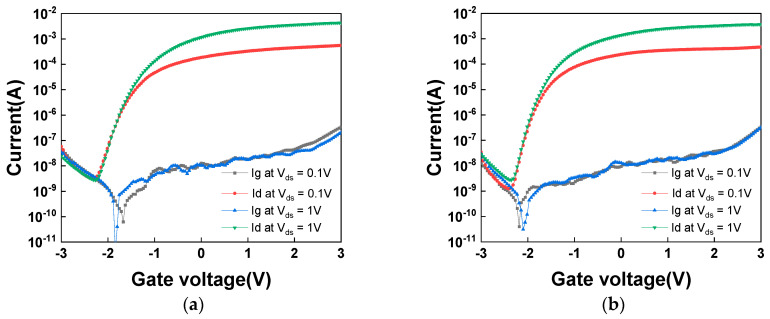
The *I_ds_*-*V_gs_* and *I_g_*-*V_gs_* characteristics of 3 nm SnO_2_ nFETs at different drain voltages (**a**) before UV annealing and (**b**) after UV annealing.

Figure 6a,b show the *I_ds_*-*V_gs_* and *I_g_*-*V_gs_* characteristics at *V_ds_* = 0.1 V and 1 V for 3 nm thick SnO*_2_* nFETs with and without UV annealing, respectively. The UV annealing improves the I_ON_/I_OFF_ from 1.9 × 10^5^ to 3.8 × 10^5^ and *SS* from 297 to 201 mV/decade. The measured *SS* is comparable to or better than those of the reported oxide–semiconductor FETs [69,70,71,72]. Remarkably, this sharp *SS* is achieved despite the presence of two oxide/semiconductor interfaces in the ultra-thin 3 nm body structure, which typically pose challenges for interface trap control. The *SS* is still above the theoretical value of 60 mV/decade. Further improvement can be reached by improving the gate dielectric stack and SnO_2_ deposition using Atomic Layer Deposition. Such improvement caused by UV annealing is attributed to the effective enhancement of the channel quality, leading to a superior electrical performance.

**Figure 7:**
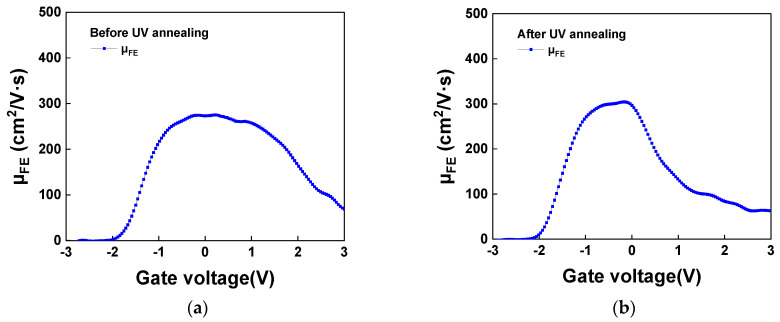
*µ_FE_*-*V_gs_* characteristics for 3 nm SnO_2_ nFET (**a**) before UV annealing and (**b**) after UV annealing.

Figure 7a,b further illustrate the *µ_FE_*-*V_gs_* characteristics at *V_ds_* = 0.1 V for devices with and without UV annealing. The extraction methods for µ_FE_ and µ_eff_ are summarized as follows [73]:

(1) $$\mu_{FE}=\;\frac{L}{W}\frac{dI_{ds}/dV_{gs}}{C_{OX}V_{ds}}\;{|}_{small\;V_{ds}}=\;\frac{L}{W}\frac{g_{m}}{C_{OX}V_{ds}}\;{|}_{small\;V_{ds}}$$

(2) $${\;\mu }_{eff}=\frac{L}{W}\frac{g_{d}}{\int_{-\infty }^{V_{gs}}C_{gs}\left(V_{gs}\right)dV_{gs}}\;{|}_{small\;V_{ds}}$$

Here, *L* and *W* denote the channel length and width, respectively, while *C_OX_* is the gate capacitance per unit area, *g_m_* represents the transconductance, *g_d_* is the drain conductance. The low-frequency *C_gs_-V_gs_* data are used for μ_eff_ calculation. Mobility plays a critical role in device performance; a higher mobility enables lower operating voltages, which in turn reduces both power consumption and operating temperature. Such improvements are particularly important for the advancement of modern AI chips. It can be observed that, after UV annealing, µ_FE_ increased from 275 to 304 cm^2^/V·s. It is important to note that the contact resistance (R_C_) causes a voltage drop across the source/drain regions, reducing the effective *V_ds_* and the measured current. Since R_C_ is a fundamental challenge for 2D-material and ultra-thin-body FETs, the reported µ_FE_ and µ_eff_ data presented here, without de-embedding R_C_, represent underestimated values. Therefore, the intrinsic channel mobility is expected to be even higher. Further R_C_ improvement can be expected by using low work-function metals [74], which are crucial for 2D materials and UTB FETs.

**Figure 8:**
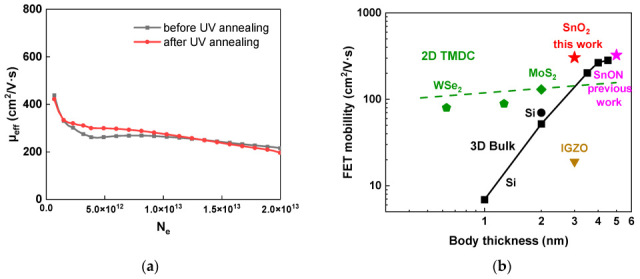
(**a**) µ_eff_-N_e_ characteristics of 3 nm SnO_2_ nFET before/after UV annealing; (**b**) FET mobility versus body thickness; data are from [1,49,55,62,75]. Our µ_eff_ values were taken at a high N_e_ of 5 × 10^12^ cm^−2^.

Figure 8a presents the typical µ_eff_ versus N_e_ characteristics before and after UV annealing. At an N_e_ of 5 × 10^12^ cm^−2^, µ_eff_ increases from 262 to 301 cm^2^/V·s after UV annealing. A significant increase in µ_eff_ is observed at an N_e_ below 5 × 10^12^ cm^−2^, which indicates suppressed charged-defect scattering [70]. Such defects are generally observed in oxide semiconductors, where adding nitrogen is one method to improve them [48,75,76,77,78]. Alternatively, as shown in this work, UV annealing can also decrease such charged oxide defects and improve mobility. Therefore, the µ_eff_ analysis provides more insight into scattering mechanism compared with µ_FE_, which is essential for FET device modeling. Figure 8b shows the µ_eff_ as a function of body thickness, where the µ_eff_ values are obtained at an N_e_ of 5 × 10^12^ cm^−2^. Such a higher N_e_ must be used since it delivers a higher output *I_ds_* and results in a faster speed for the integrated circuit (IC). At a body/channel thickness of 3 nm, the proposed device achieves a notably higher µ_eff_ mobility compared with other channel material technologies, including 2D MoS_2_ and WSe_2_, and even more than 2 times higher than single-crystal Si used for sub-1 nm node IC manufacturing [1,48]. The SnO_2_ nFET exhibits a significantly higher mobility compared to other oxide semiconductors, primarily due to the larger radius of the Sn 5 s orbitals, which enhances orbital overlap, facilitates electron transport, and reduces the effective mass [79,80]. The enhanced µ_eff_ is strongly associated with the small effective mass [48] and higher-κ value [75] of SnO_2_. The former is similar to the mechanism responsible for the higher mobility of ultra-thin-body Ge FETs compared with Si FETs [3]. In particular, SnO_2_ possesses a direct energy bandgap with an effective mass of 0.41 m_e_*, which is considerably smaller than that of Si, thereby enabling superior carrier transport properties in the ultra-thin channel. As the body thickness is scaled down, the electron wave function spreads throughout the entire channel, making body defects as critical as interface defects in determining carrier mobility. The strategies to overcome this limitation are to employ materials with a smaller effective mass [3], improved gate dielectric/semiconductor interfaces, and decreased channel defects. The significantly higher-κ value decreases the effective electric field in the SnO_2_ channel and improves the µ_eff_ [75]. Compared with previous work on 5 nm thick SnON FETs [75], the mobility of the present 3 nm thick SnO_2_ devices does not exhibit a noticeable degradation despite the reduced channel thickness. This result shows that the UV annealing can effectively suppress interface and body traps, thereby contributing to the preserved high mobility [68]. In addition, unlike 2D materials that require complex transfer processes onto 12-inch wafers, the 3 nm SnO_2_ channel in this work can be directly fabricated on a 12-inch Si substrate via a simple sputtering process, thereby greatly reducing fabrication costs.

Table 2 summarizes the UV annealing effects on 3 nm thick SnO_2_ FETs. The gate–dielectric/semiconductor interface trap density (D_it_) can be calculated from *SS* [81]:

(3) $$D_{it}=\left(\frac{qSS}{k_{B}Tln\left(10\right)}-1\right)\frac{C_{ox}}{q}$$

The D_it_ is 1.6 × 10^13^ cm^−2^·eV^−1^ for devices without UV annealing, which was reduced to 9.8 × 10^12^ cm^−2^·eV^−1^ after UV annealing. X-ray photoelectron spectroscopy (XPS) was used to understand the UV annealing improvement [82]. From XPS measurements, the peak intensity of Sn^4+^ increases and unwanted Sn^4+^ decreases after UV annealing. The mechanism of UV annealing can be summarized as follows:

(4) $$O_{2}+hv\left(185\;nm\right)\to 2O,\;O+O_{2}\to O_{3\;},\;{\;O}_{3}+hv\left(254\;nm\right)\to O_{2}+O^{*}\;$$

The mechanism relies on the superior reactivity of generated excited oxygen radical O* compared to ground-state O_2_ and O. These O* species efficiently diffuse into the film to repair oxygen vacancies and promote Sn–O bond formation. Crucially, the process reduces the concentration of unstable Sn^2+^ states, which possess active dangling bonds and behave as p-type semiconductors [83] that may degrade n-type device reliability. By converting these unstable Sn^2+^ species into the stable Sn^4+^ phase, UV annealing therefore minimizes charge trapping sites. This in turn enhances overall device characteristics and may lower the potential reliability issues under bias-temperature stress. This reduction in defect density is consistent with the healed interface, and the significant enhancement in µ_FE_ and µ_eff_ shown in Figure 8a. Consequently, UV annealing simultaneously improves all critical device parameters, including µ_FE_, µ_eff_, *SS*, D_it_, and I_ON_/I_OFF_.

**Figure 9:**
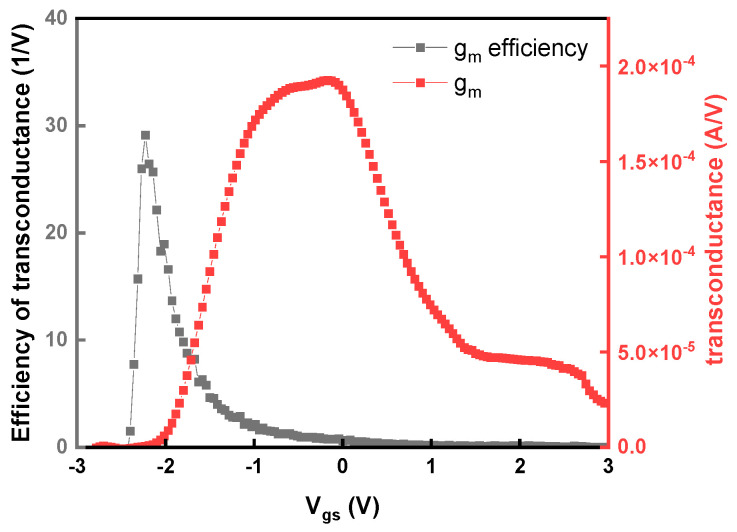
The transconductance and transconductance efficiency of UV-annealed 3 nm T_body_ SnO_2_ FET.

Figure 9 shows the transconductance (g_m_) and transconductance efficiency (g_m_/I_D_) versus V_gs_ of UV-annealed 3 nm T_body_ SnO_2_ FET. The device exhibits a remarkable peak g_m_/I_D_ value of 30 V^−1^. This value is close to the theoretical limit of 38.5 V^−1^ (q/kT) at 300 K, indicating an excellent FET performance from UV annealing. The high mobility allows the device to offer a superior current drive and excellent efficiency in the low-power regime.

**Figure 10:**
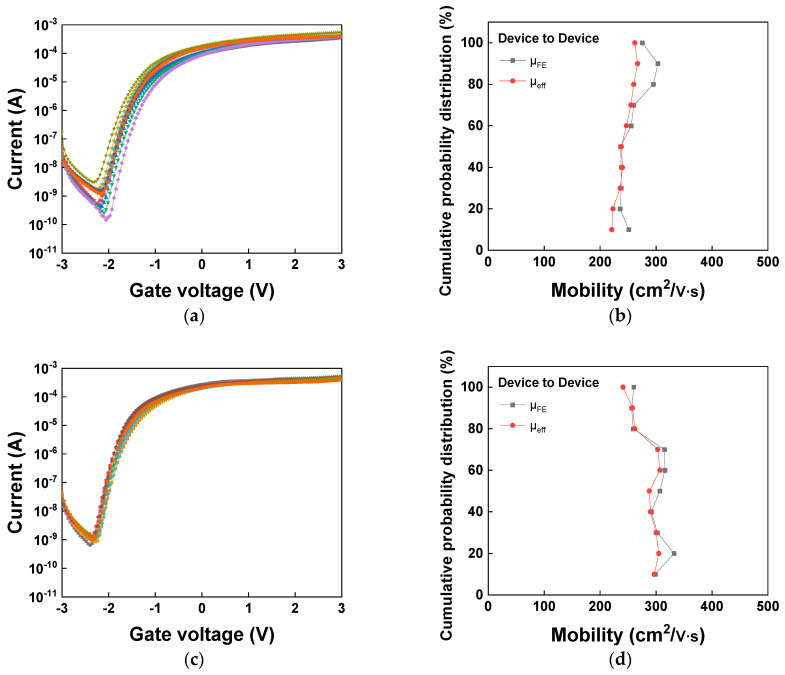
*I_ds_*-*V_gs_* characteristics of (**a**) device-to-device and (**b**) µ_FE_ and µ_eff_ distributions for 3 nm SnO_2_ nFETs before UV annealing and (**c**,**d**) after UV annealing.

To further ensure both device and mobility reproducibility, Figure 10 presents the *I_ds_*-*V_gs_* characteristic distributions along with the of µ_FE_ and µ_eff_ distributions. Table 3 presents the statistical data of key parameters derived from 10 top-performing devices selected from a batch of 32. The post-UV annealed devices exhibit significantly improved performance and uniformity compared to the pre-annealed ones. The µ_eff_ value is at a high N_e_ of 5 × 10^12^ cm^−2^. These results demonstrate that most devices exhibit a consistent performance, confirming the reproducibility and reliability of the proposed process.

## 4. Conclusions

An excellent device integrity of µ_FE_, µ_eff_, *SS*, D_it_, and I_ON_/I_OFF_ is achieved in 3 nm quasi-2D SnO_2_ nFETs. The record high µ_eff_ at a 3 nm channel thickness is due to the UV annealing improving the gate–dielectric/SnO_2_ interface property and SnO_2_ body defects. Moreover, the simple fabrication process is fully compatible with existing IC fabs for large-scale production. Such advancements are highly beneficial for realizing low-power IC and monolithic 3D integration.

## Figures and Tables

**Table 2:** Performance comparison of the 3 nm SnO_2_ nFETs before/after UV annealing.

3 nm SnO_2_ FET	µ_FE_ (cm^2^/V·s)	µ_eff_ (cm^2^/V·s) @ 5 × 10^12^ cm^−2^	*SS* (mV/Decade)	D_it_ (cm^−2^·eV^−1^)	I_ON_/I_OFF_
Before UV anneal	275	262	297	1.6 × 10^13^	1.9 × 10^5^
After UV anneal	304	301	201	9.8 × 10^12^	3.8 × 10^5^

**Table 3:** Mean and standard deviation (σ) for key parameters before and after UV annealing.

	µ_FE_ (cm^2^/V·s)	µ_eff_ (cm^2^/V·s)	*SS* (mV/Decade)	V_T_
Before UV				
Mean	258	244	267.5	−1.58
σ	23.6	15.2	16.7	0.089
After UV				
Mean	294	285	211	−1.84
σ	25.3	21.9	20.8	0.056

## Data Availability

The data presented in this study are available on request from the corresponding author. The data are not publicly available due to privacy.
